# The Medical Student Summer Research Program at the University of Texas Medical Branch at Galveston: building research foundations

**DOI:** 10.1080/10872981.2019.1581523

**Published:** 2019-03-04

**Authors:** Lisa Cain, George Kramer, Monique Ferguson

**Affiliations:** a Department of Diagnostic and Biomedical Sciences, School of Dentistry, University of Texas Health Science Center at Houston, Houston, TX, USA; b Department of Anesthesiology, University of Texas Medical Branch at Galveston, Galveston, TX, USA; c Department of Internal Medicine, Division of Infectious Diseases, University of Texas Medical Branch at Galveston, Galveston, TX, USA

**Keywords:** Medical student summer research program, curriculum-based research programs, medical education, physician scientist careers, medical student research training opportunities

## Abstract

**Background**: Interest in incorporating research into the medical school curriculum has grown over the years. One of the challenges involved with providing research to medical students is developing programs that allow a large number of students to perform research. This involves securing faculty to mentor students in the design of research projects. In order to accommodate students with research interests, well-established research programs must be implemented.

**Objective**: This article describes the design and implementation of a curriculum-based research program for medical students at the University of Texas Medical Branch (UTMB) at Galveston. The main objective of this article is to describe the program for the purpose of assisting other medical schools to develop a similar student research program.

**Design**: At UTMB we established a Medical Student Summer Research Program (MSSRP) that occurred between the first year and the second year of medical school. Between the years 2000–2017, MSSRP accommodated a minimum of 39 and a maximum of 90 students during an 8 week period. Two surveys were conducted to collect students’ views on how MSSRP affected their interest in research. We performed a proportion statistical analysis on the data from both surveys in order to determine the significance of the responses.

**Results**: The benefit of MSSRP is that it provided medical students with an exposure to research. According to the proportions test, the responses were statistically significant with 85% of 26 third and fourth year students stating they would continue to incorporate research into their medical careers; 75% stating that MSSRP increased their interest in research; and 85% responding that MSSRP helped them to understand research methodology.

**Conclusions**: MSSRP is a curriculum-based program that provides a framework to other medical institutions interested in the development of similar student research programs and provides students the exposure and option to continue with research as a component of their medical profession.

## Introduction

The American Association of Medical Colleges (AAMC) 2014 Medical School Graduation Questionnaire cited a 7.9% increase in the proportion of students who participated with scientific research projects under the supervision of a faculty mentor between 2010 and 2014. ^[1]^ This indicates that more medical schools are incorporating research opportunities for medical students into their curriculum. The major challenge is providing research opportunities for a large number of medical students at a single institution. The establishment of research programs for medical students helps them to develop critical thinking skills in evaluating scientific evidence to prepare for competitive residency applications. In addition, it introduces the concept of research as a possible component of their medical careers, helps to establish self-directed lifelong learning, and emphasizes the importance of literature searches and analytical thinking [2,3]. Student research programs are presented in the form of summer programs, elective courses, research tracks, and four year supplemental programs. [4–7] They are supported by intramural and extramural funding, endowments, and alumni associations. Students may also enroll in a dual M.D.-Ph.D. degree program. Due to the recent decline in the number of clinical scientists [8–15], emphasizing the importance of the transition from research to clinical discovery is pertinent to a medical student’s knowledge base. The main outcomes of a research experience are the assimilation of research techniques that can be utilized to pursue additional research training opportunities or to pursue research as a component of their medical profession. [2,16,17] Students with research experience who do not pursue research as a career can still apply these tools to clinically relevant problems and use the skills to develop an evidence-based approach to patient care. Providing research opportunities for students also emphasizes the connection between basic, clinical, and translational research. Therefore, providing medical students with opportunities for research is an essential tool for career development.

The University of Texas Medical Branch (UTMB) at Galveston has a productive history of creating research opportunities for medical students, with an emphasis on the relationship between research and medicine. An example of such an opportunity is the Medical Student Summer Research Program (MSSRP), an elective course that is a part of UTMB’s integrative medical school curriculum. After the successful completion of their first year, UTMB medical students can participate in MSSRP for eight weeks during the summer between years one and two. Although not a required course, students receive two elective credits for performing a research project under the supervision of a UTMB faculty. The MSSRP provides formal research training for UTMB medical students. Since the year 2000, 916 UTMB medical students have participated in research (Table 1). Although the majority of the students performed research on UTMB’s campus, with application approval by the MSSRP director, some are allowed to conduct research at other academic institutions under the supervision of a faculty member. As MSSRP expanded, translational and educational research were included as categories during the years 2014–2017.

**Table 1.** MSSRP student participants 2000–2016.

Year	Total number of student participants	Number of abstracts submitted	Clinical Science Research Abstracts	Basic Science Research Abstracts	Translational Science Research Abstracts	Educational Science Research Abstracts	Number of posters presented
2000	40	40	15	25			40
2001	45	45	27	18			45
2002	42	42	21	21			42
2003	38	38	12	26			38
2004	54	40	17	37			54
2005	43	41	14	29			43
2006	37	37	17	20			37
2007	58	58	24	34			58
2008	40	40	15	25			40
2009*	0	0	0	0			0
2010	56	56	25	31			56
2011	61	61	35	26			61
2012	52	52	32	20			52
2013	68	68	35	33			68
2014	90	90	37	32	18	3	90
2015	56	56	24	21	11	0	56
2016	77	77	39	18	14	6	77
2017	59	59	17	21	18	3	59
**Total**	**916**	**916**	**406**	**437**	**61**	**12**	**916**

*In 2009 UTMB was recovering from the effects of hurricane IKE therefore MSSRP was not held.

## Organization of the MSSRP program

### Personnel

The MSSRP program is administered by the Office of Student Affairs and Admissions at UTMB. It is an 8 week program scheduled during the summer from May to June. It is organized and implemented by the program director, two faculty co-directors, and a program coordinator. The program director facilitates the organization of the program infrastructure, interacts with program collaborators, assigns grades, mentors medical students and manages budgetary matters. The two faculty co-directors assist with mentoring medical students, facilitate mid-term presentations, assign poster judges, facilitate workshop for poster preparations, and serve as judges during posters/awards ceremony. The program coordinator organizes student registration, arranges the program schedule, and coordinates the logistics of meeting rooms for all program activities. The program expenses, that essentially cover the cost of the poster session, are funded by the Office of Student Affairs and Admissions.

### Registration and selection of medical students

UTMB School of Medicine (SOM) admits approximately 230 students annually to medical school. Over the years, student participation in MSSRP has ranged from 16–39%. Students receive information about the program during an annual campus wide electives preview sponsored by the Office of Clinical Education; then MSSRP directors and program coordinator host a special information meeting for all students interested in performing research during the summer. The information meeting provides students with program details followed by research presentations facilitated by invited faculty members.

Registration for the program involves completing a registration form that includes the student’s name, the date of the research, and the name of the designated faculty mentor and an abstract of the research plan. The form must be signed by the faculty member who will supervise the student’s research and the MSSRP director. Students have the option to register for a 4 week Medical Student Summer Research Elective (MSSRE), if they choose not to participate during the 8 week elective. However, MSSRE is only for students who due to circumstantial reasons cannot perform the 8 week elective, and approval to participate must be granted by the MSSRP director. All MSSRE students are required to complete a registration form; however, these students are exempt from participating with the MSSRP poster session and awards ceremony. Scheduled meetings, a brief written research report, and an oral presentation are the required components of this program. MSSRE students are required to write a report of their research experience or to present a PowerPoint presentation.

The MSSRP director and co-directors provide assistance in matching students with research faculty mentors on UTMB’s campus – and with other internal medical student research programs that provide stipend support. All students must meet the deadline to register. The registration forms are submitted to the registrar’s office and the final pass/fail grade is assigned at the completion of the program by the MSSRP director (see Student Evaluations). Programs such as the Translational Research Track, the NIH infectious Diseases Student Research Training Program, and the Sealy Center on Aging Research Program enroll their students in MSSRP and provide additional didactic content during the summer. Faculty mentors are responsible for assigning the student’s ‘pass’ or ‘fail’ grades.

### Selection of faculty mentors

The MSSRP provides a positive and productive experience for students while engaging faculty who desire to train a medical student in their lab. Over the years MSSRP has been an enriching experience for both the students and faculty members. Approximately five months prior to the start of MSSRP, the program coordinator disseminates a faculty invitation and request letters in order to secure faculty participants. Research faculty respond by stating (in writing) their desire to accommodate a medical student in their laboratory during the summer. They indicate the number of students they can accommodate and also submit an abstract of their own research. The program coordinator collects the abstracts and organizes them into an institutional database. This information is placed online (e.g., Blackboard) for easy student access. When selecting a research faculty, medical students go to the online database, search the internet for faculty in specific interest areas, or obtain referrals from other students. Students then contact the faculty members and express their interest to work in their laboratory. If a student has difficulty contacting a faculty member or identifying a mentor, the MSSRP program director will assist. The student’s faculty mentor is expected to pay the costs for the student’s poster, however, there is no salary or stipend requirement imposed on the faculty mentor unless the students are part of a funded program that provides a stipend. At the conclusion of 8 weeks, students are required to complete a post-survey inquiring about their research experience and future interest as a component of their medical profession. A pre-survey is also administered at the beginning of the program to determine the student’s insight regarding research.

### MSSRP program components

The components of the program include an orientation, daily laboratory research, mid-term small group reports, a poster session workshop, and a final poster session and awards ceremony.

#### Learning objectives

MSSRP is a curriculum-based research program for medical students who have completed their first year of medical school at UTMB. At the conclusion of the program medical students will be able to identify and explain research methodology and protocols; recognize and distinguish between a research laboratory and an investigative environment; students will develop and exercise the concept of collaborative scientific teamwork; as well as manage and produce a scholarly product to present in a public forum.

#### Orientation

Orientation is scheduled on the first day of the program and consists of a keynote speaker whose purpose is to get the students excited about research and to share their research experiences. The director of the Honors Research Program is also invited to talk to the students. This is a very important presentation during orientation because for more than 25 years, the UTMB Honors Research Program has provided research opportunities for medical students which augment the regular medical school curriculum and leads to the conferment of Honors in Research in a specialized field of research. The Honors Research Program director approves research proposals and a full-time faculty member oversees project performance. Students in the MSSRP program may consider continuing their research with their mentor by enrolling in the Honors Research Program. **Honors are conferred on student at graduation pending completion of a submission ready manuscript and successful defense of their research before a faculty panel**. During orientation students are also provided with laboratory safety technique training by the environment health and safety director.

#### Laboratory participation and mid-program reports

The MSSRP is a highly- structured program, supplemented by individual laboratory requirements that students are required to fulfill such as attending laboratory meetings. Students are expected to perform research on a daily basis for the full 8 weeks. If they perform research at an external institution, they must return to UTMB to participate in the poster session at the conclusion of the program. The individual research mentors may also have separate laboratory activities in which the students are required to participate. Student research projects may include a variety of disciplines such as basic science, clinical, translational, education and humanities research. Over the years as indicated in Table 1, the number of students participating in translational and educational research has increased.

Midway through MSSRP (the completion of 4 weeks of research), a mid-term small group facilitation activity is held. This midterm activity consists of a faculty keynote speaker who provides essential information on the overall expectations of the research process. Following the keynote presentation MSSRP, students separate into 6 to 8 small groups. Each small group is assigned a research faculty who serves as a facilitator. Individual students present their research to the group, share what they have learned, and discuss any challenges that they have encountered. The small group meeting allows the students to gain effective feedback on their progress from the facilitators and their peers, and identifies any student who might have encountered challenges during their research experience. The few difficulties students have faced over the years involve issues in getting protocol approval, research setbacks, or in rare cases a lack of close supervision and guidance from their faculty research mentors. In these cases, the student contacts the program directors to receive advice on how to navigate forward.

#### MSSRP poster session & awards ceremony

The 8 week research program concludes with a poster session and an awards ceremony. Students must complete the poster session in order to receive a grade of ‘pass.’ Two weeks prior to the final ceremony, the faculty MSSRP co-directors conduct a poster session workshop to provide instructions on how to prepare and present a poster. This workshop is useful, specifically for students who are performing research for the first time.

Before the end of MSSRP (at 4 weeks), a request for judges is distributed by the program coordinator. The faculty directors meet and assign each student three judges. The poster session occurs on the last day of the program and includes a keynote speaker with a productive research record. Administrators, faculty members, and students are invited to attend the poster session which is followed by an awards ceremony. During the awards ceremony, all MSSRP students are presented a certificate for their participation in MSSRP. In addition, monetary awards are presented to students with outstanding posters in various categories. These awards are generously funded by various departments and faculty at UTMB.

### Student evaluations and program results

The students must complete all program components in order to receive their grade. At the end of the program, research faculty mentors are required to submit a student evaluation form in order for student participants to receive a ‘pass/fail’ grade.

Students are also given a pre- and post survey to determine the impact of the MSSRP on their interest in research. The results were tabulated for each year for both the pre and the post survey and the mean percentile of the three years (2014, 2015 & 2016) for each question was tabulated. In Table 1, 100% of students who participated in years 2014–2016 responded to the pre and post survey in Table 2. As such, the pre and post survey in Table 2 consists of a total of 223 students; data was obtained from MSSRP participants in 2014 (90 students), 2015 (56 students) and 2016 (77 students).

**Table 2.** MSSRP student survey 2014–2016 the average mean of percentiles (Neutral values were not included).

Questions Total # of student participants = 223	Strongly Agree/Agree Pre – Survey*	Strongly Agree/Agree Post – Survey*	Strongly/Somewhat Disagree Pre-Survey*	Strongly/Somewhat Disagree Post-survey*
Clinical Research is vital to the future of quality health care	98.3%	97.4%	0%	2.4%
Basic research is vital to the future of quality health care	97%	96.0%	0%	2%
Participation in Research is an integral part to being a physician	73%	75%	5.2%	7.2%
I will probably not be involved in research to any appreciable extent during my career	13.3%	19%	59%	58.4%
I will probably devote 1/3 to ½ of my career to research	25.1%	24%	30%	29.3%
I will probably devote the majority of my time to research during my career	8.4%	7%	63%	63%

*Proportion analysis test was applied comparing ‘strongly agree/strong disagree’ for pre and post survey and was determined not be statistically significant, p-value > 0.05. Statistical analyses were performed using R statistical software (R Core Team 2017) [18].

The most appropriate test for analyzing the significance of the data collected in Tables 1 & 2 is a proportion statistical test [18]. In Table 2 the test was used to assess if the proportion that ‘agreed’ or ‘disagreed’ differed between the pre and post survey. The pre and post surveys (Table 2) indicated that the majority (96–98%) of the student across all three years strongly agreed that both clinical and basic science research was vital to the future of quality healthcare. Only a few students, 2.0% and 2.4%, changed their opinions after performing research. The majority of the students, 73% and 75% respectively, in the pre and post survey felt that participating in research is integral to being a physician. In regards to making research an active component of their career, 25% and 24% respectively of the students in the pre and post survey strongly agreed that they would commit one-third to one-half of the time in their career to research. However, the majority 59% and 58% in the pre and post survey strongly disagreed with the statement that they would not be involved in research during their career and 63% strongly disagreed in the pre and post survey with the statement that they would devote the majority of their career to research. Despite the differences in the responses to the pre and post survey, according to the proportions test, it was determined that there was no statistical difference between the pre and the post survey data.

An additional survey was completed by 26 third- and fourth year medical students who completed MSSRP at the end of their first year in medical school. This survey examined the benefits of MSSRP. To analyze the data in Table 3 we used the proportion test to assess if the number of participants responding ‘yes’ differed from the null hypothesis of 50%. The major benefits as indicated in Table 3 were present in the fact that 85% of the students stated that they would continue to incorporate research into their medical career; 75% of the students stated that MSSRP increased their interest in research; and 85% of the students stated that MSSRP helped them to understand research methodology. According to the proportions test the responses were statistically significant.

**Table 3.** Questionnaire for post MSSRP participants (26 participants).

Question	Yes	No
I have published a research paper or papers since completing MSSRP	44%	56%
I have presented at a national conference since completing MSSRP	42%	58%
I have presented at a local or regional conference since completing MSSRP	62%	38%
I am still involved in research since completing MSSRP	62%	38%
MSSRP was responsible for motivating me to continue to be involved in research	69%	31%
I had no experience in research until I participated in MSSRP	50%	50%
I will continue to incorporate research into my medical career	85%**	15%**
MSSRP increased my interest in research	77%**	23%**
MSSRP helped me to better understand research methodology	85%**	16%**

Proportion analysis test was applied to assess if the number of participants responding ‘yes’ differed from the null hypothesis of 50%. Statistical analyses were performed using R statistical software (R Core Team 2017) [18]. **Data was determined to be statistically significant, with p-value < 0.05.

The majority of the students (62%) also indicated that they were successful in presenting at a local and regional conferences and that they are still involved in research. Since completing MSSRP 44% of the students have published a research paper. It is unreasonable to expect that every student will produce a paper due to the fact that MSSRP only exists for 8 weeks. Most of the time a manuscript involves student efforts beyond the summer program. The students who completed the survey articulated that MSSRP was valuable to their professional development.

## Limitations

This is a description of a curriculum-based medical student research program, and not an evaluation study. However, we submitted two surveys in order to obtain the opinion of students on how MSSRP influenced their interest in research. Various limitations were identified with these surveys including, the lack of respondents to the questionnaire of post MSSRP participants in Table 3 (with only 20% responding) and the limited scope of our survey questions in the pre and post surveys in Tables 1 & 2. For future survey development, we will broaden the scope of our questionnaires and increase the sample size of survey participants. Despite these limitations, MSSRP provides students the opportunity to gain a realistic view of what it is like to perform research and provides the exposure that will allow them to decide if they want to continue with research as a component of their medical profession.

## Conclusions

MSSRP is a well-established research program at UTMB that describes the participation of 916 medical students during the years 2000–2016 (Table 1). To date, this is the first published report that describes extensively a curriculum based well-structured elective for medical students of this magnitude; MSSRP serves as a model to other medical schools interested in the development of similar student research programs. Additionally, MSSRP provides students the opportunity to gain a realistic perspective of what it is like to perform research, and allows them to decide if they want to make research a component of their career path. As indicated in Table 2, the majority of the students, 73% pre survey and 75% post survey felt that being involved in research was integral to their career as a future physician. It is also important to note that 97–98% of the students felt that basic science and clinical research was important to healthcare. On the other hand, the survey indicated that the majority (63%) of the students felt that research is not their primary focus and would not commit a significant amount of time to performing research. This was expected, as students who participated in the MSSRP had no previous experience in research and did not have a realistic idea of what was involved. Therefore, we predicted that some students would turn away from research after being exposed as indicated by a slight decrease in research interest shown in the post surveys (Table 2). The survey data analysis simply provided an awareness of how the students felt about their research experience, and was not meant to be an in depth evaluation of the program. It is also worth noting that students had an equal respect for basic science and clinical research (Table 2). Although the survey did not query translational research, this would be an interesting question to incorporate into future surveys. In Table 3, a survey completed by 26 (20%) third and fourth post MSSRP participants (who participated in years 2015 and 2016), provided additional evidence that MSSRP provided a positive impact in regards to increasing the interest of students (77%) in research; and to integrate research as part of their career path (85%). According to the proportions test the responses indicated above were statistically significant.

Students who participated in MSSRP have used the experience to become more competitive for residency programs, to increase their knowledge of research, to produce publications, and to present at regional scientific conferences. Overall, MSSRP represents a platform for the development of curriculum-based research programs at other medical school institutions, with the hope of increasing the exposure of students to research and in producing more physician scientists in the near future.
